# Impact of Post-Annealing Treatment on the Microstructure, Recrystallization and Mechanical Behavior of Hot-Rolled Mg-Al-Zn-Ca Alloy

**DOI:** 10.3390/ma18214897

**Published:** 2025-10-26

**Authors:** Arasappan Rajesh Kannan, Hafiz Muhammad Rehan Tariq, Muhammad Ishtiaq, Ha-Seong Baek, Umer Masood Chaudhry, Tea-Sung Jun

**Affiliations:** 1 Department of Mechanical Engineering, Incheon National University, Incheon 22012, Republic of Korea; rajeshkannan@inu.ac.kr (A.R.K.); 202221057@inu.ac.kr (H.M.R.T.);; 2 Research Institute for Engineering and Technology, Incheon National University, Incheon 22012, Republic of Korea; 3 Department of Materials Engineering and Convergence Technology, Gyeongsang National University, Jinju 52828, Republic of Korea

**Keywords:** Mg alloy, post-annealing treatment, texture, mechanical properties, particle stimulated nucleation (PSN)

## Abstract

Lightweight magnesium alloys are gaining increasing attention as potential structural materials for automotive and aerospace applications due to their high specific strength and excellent recyclability. However, their formability and mechanical performance are often limited by strong basal texture and limited recrystallization during thermomechanical processing. In this context, the present study systematically investigates the effect of post-annealing treatment on the microstructural evolution, recrystallization behavior, and mechanical response of a hot-rolled Mg-3Al-1Zn-1Ca alloy. Detailed microstructural characterization revealed that Al_2_Ca precipitates were uniformly distributed along grain boundaries in the as-received (AR) condition, where they contributed to significant pinning of boundary migration. Post-annealing treatment (350 °C, furnace cooling) resulted in non-uniform grain coarsening, driven by the interplay of precipitate pinning and differential stored strain energy, while also facilitating particle-stimulated nucleation (PSN) and recrystallization. Electron backscatter diffraction (EBSD) analysis confirmed a substantial increase in the fraction of high-angle grain boundaries and recrystallized grains in the heat-treated (HT) state, with kernel average misorientation (KAM) and grain orientation spread (GOS) analyses indicating pronounced recovery of lattice distortions. Mechanical testing demonstrated a significant decrease in yield strength (263 MPa to 187.4 MPa) and hardness (65.7 to 54.1 HV) due to dislocation annihilation and stress relaxation, while ultimate tensile strength remained nearly unchanged (~338 MPa) and ductility improved markedly (12.6% to 16.4%). These findings highlight the dual role of Al_2_Ca precipitates in promoting recrystallization through PSN while simultaneously restricting excessive grain growth through Zener pinning.

## 1. Introduction

Magnesium (Mg) alloys are gaining attention in the transportation industry for their potential to reduce vehicle weight [1,2]. However, their widespread use faces challenges because, compared to other structural materials, Mg alloys have lower specific strength and show difficulties in formability and workability due to their hexagonal close-packed (HCP) crystal structure [3,4]. While Mg has multiple slip systems, high critical resolved shear stress (CRSS) values hinder effective activation, leaving only basal slip systems operational at room temperature [5,6]. This inadequacy in uniform deformation is compensated for by twins, which accommodate plastic strain during deformation.

The AZ series of Mg alloys stands out as the most prevalent and economically viable option among commercially available Mg alloys. As outlined in the Mg-Al binary phase diagram [7], the equilibrium β-phase constitutes second-phase precipitates, characterized by Mg_17_Al_12_. These secondary phase particles exert varying effects on microstructure and texture evolution throughout thermomechanical processing, with their impact depending upon factors like size and distribution within the material [8]. However, the β-phase is intrinsically brittle and thermally unstable, often accelerating crack initiation and limiting ductility during service. To overcome these challenges, alloying strategies have been explored to suppress the formation of β-Mg_17_Al_12_ and instead promote the development of more stable intermetallic compounds. Rare-earth (RE) additions have been shown to effectively refine grains, weaken basal textures, and enhance ductility by consuming Al and forming Al-RE phases [9]. Nonetheless, the high cost and limited supply of RE elements restrict their application in large-scale production. Calcium (Ca) has emerged as a cost-effective alternative, reacting with Al to form thermally stable Al_2_Ca particles that impede dislocation motion and grain boundary migration, while also reducing reliance on β-Mg_17_Al_12_ [10,11]. During hot rolling, these Ca-containing precipitates alter the CRSS by elevating the resistance to basal slip and twin propagation while facilitating non-basal slip activity [12]. Although this shift in deformation mechanisms helps to weaken basal texture, the rolling process still introduces significant strain heterogeneity and internal stresses into the microstructure. To stabilize the beneficial effects of Ca additions and further optimize the balance between strength and ductility, post-deformation annealing becomes essential. This process relieves internal stresses, modifies the microstructure, and enhances precipitation strengthening [13]. For instance, Chaudry et al. reported that annealing of as-rolled AZ61-CaO alloys promoted the formation of (Mg, Al)_2_Ca precipitates, suppressed twinning, and significantly improved the tensile strength through precipitation hardening [14]. In Mg-Al-Zn alloys containing secondary phases, annealing after thermomechanical processing has also been shown to accelerate particle-stimulated nucleation (PSN), facilitating recrystallization and reducing the dominance of basal textures, which in turn leads to more randomized grain orientations and improved formability [15].

So far, systematic studies on the microstructural and mechanical response of Mg-Al-Zn-Ca alloys subjected to the post-annealing treatment remain limited. Therefore, it is essential to investigate how annealing influences the microstructure, texture development, and mechanical deformation behavior of such alloys. In this work, a Mg-3Al-1Zn-1Ca (by wt.%) alloy plate produced by hot rolling was examined in both as-received and post-annealed conditions. Detailed microstructural characterization was conducted to analyze the morphology, volume fraction, and crystallographic features of intermetallic phases. Microhardness and tensile tests were performed to evaluate the mechanical response. This comprehensive approach enables a deeper understanding of the interplay between heat treatment, microstructure, and mechanical properties in Mg-Al-Zn-Ca alloys.

## 2. Materials and Methods

A 1 mm thick Mg-3Al-1Zn-1Ca alloy plate, produced by hot rolling, was obtained from POSCO, Pohang, South Korea. The alloy was further heat-treated at 350 °C in the Muffle furnace (AWF-12/25-Lenton, Hope, Hope Valley, UK), followed by furnace cooling with a cooling rate of 30 °C/min. Hereafter, this sample will be called as HT specimen. For microstructural analysis of both the as-received (AR) and HT specimens, samples were sectioned into 10 mm × 5 mm pieces from the rolling direction-transverse direction (RD-TD) plane. The specimens were cold-mounted to preserve structural integrity and then subjected to the standard metallographic preparation procedure mentioned elsewhere [16]. To characterize the distribution and morphology of intermetallic particles, scanning electron microscopy (SEM, JSM-7800F, JEOL, Tokyo, Japan) equipped with energy-dispersive X-ray spectroscopy (EDS) was employed. The particle fraction in the as-rolled (AR) specimen was quantified using ImageJ software (1.53t). The micrographs were converted to a 16-bit image type before analysis. Particle segmentation was performed using a fixed threshold range of 128–65,535 to distinguish the secondary-phase particles from the α-Mg matrix. The particle fraction and size distribution were then measured based on the binarized images obtained after thresholding. Microstructure and texture evolution of the AR and HT specimens were further investigated using electron backscatter diffraction (EBSD) in an SEM (SU-500, Hitachi, Tokyo, Japan) equipped with an EBSD system (Velocity^TM^ Super, EDAX, Mahwah, NJ, USA). EBSD scans were performed over an area of 300 µm × 300 µm with a step size of 0.5 µm, and the data were analyzed using OIM 8.6 software.

Microhardness measurements were conducted at 10 different points on the RD-TD plane using a micro-Vickers hardness tester (Shimadzu HMV-G series, Kyoto, Japan) under a 1.96 N load and a 5 s dwell time. For tensile testing, dog bone-shaped specimens were prepared from the hot-rolled sheets via water jet cutting, following ASTM E8 standard [17] dimensions, with a thickness of 1 mm and a gauge length of 25 mm. Tests were conducted at room temperature (~24 °C) using a universal testing machine (UTM, RB 301 UNITECH-T, R&B, Daejeon, South Korea) at a strain rate of 1 × 10^−3^ s^−1^. An axial extensometer (Model 3542, Epsilon Tech., Jackson, WY, USA) was attached to the gauge section to accurately record strain.

## 3. Results

### 3.1. Microstructure and Texture Evolution

**Figure 1:**
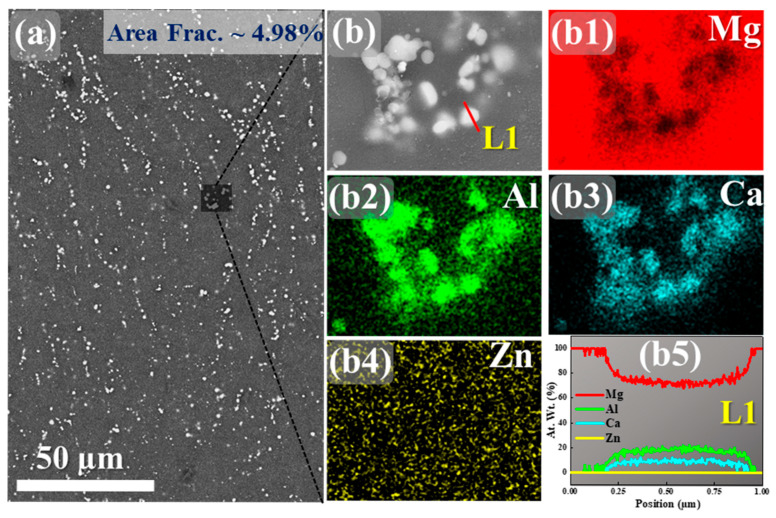
SEM micrographs and EDS analysis of secondary phase particles in the Mg-Al-Zn-Ca alloy: (**a**) BSE image showing the distribution of second-phase particles. (**b**) Magnified SEM images of the selected regions in (**a**), highlighting the morphology of the particles. (**b1**–**b4**) Corresponding EDS elemental mapping of Mg, Al, Ca, and Zn, showing enrichment of Al and Ca in the second-phase regions. (**b5**) EDS line-scan profiles (L1) across representative particles, confirming elemental segregation.

Figure 1 presents a detailed SEM-EDS analysis of the bright particles dispersed within the α-Mg matrix and their distribution in the hot-rolled Mg–Al–Zn–Ca alloy.

The backscattered electron (BSE) image (Figure 1a) shows bright contrast particles uniformly dispersed in the Mg matrix with an area fraction of ~4.98%, while the magnified view (Figure 1b) highlights their irregular morphology, typical of solidification-induced segregation. EDS elemental mapping (Figure 1(b1–b4)) highlights significant enrichment of Al and Ca in the particles with corresponding depletion of Mg, whereas Zn remains relatively uniform in the matrix, indicating limited participation in the secondary phases. This clearly indicates the role of Zn as a solid solution-strengthening element rather than a constituent of the secondary phases. The EDS line scan across a representative particle (Figure 1(b5)) further confirms compositional segregation, with distinct peaks of Al and Ca and a dip in Mg, supporting the identification of Al-Ca-rich intermetallics such as Al_2_Ca or (Mg, Al)_2_Ca, which are widely reported in the literature as stable, high-melting compounds [18]. The average size of precipitates was measured as 0.72 µm. The Al_2_Ca intermetallic phase in Mg alloys is reportedly aligned along the basal plane of the α-Mg matrix [19]. This orientation is due to the crystallographic compatibility between the Al_2_Ca phase and the α-Mg matrix, facilitating coherent precipitation [20].

These intermetallics are known to preferentially form at grain boundaries, enhancing creep resistance and thermal stability in Mg alloys, while Zn is generally retained in solid solution or forms Mg-Zn phases at higher contents [21]. The higher concentration of Ca and Mg reveals the formation of the Al_2_Ca intermetallic phase, which consumes Al solute from the Mg matrix. Given that the atomic radius of Al (1.43 Å) is smaller than that of Mg (1.60 Å), the reduction in Al content results in a decrease in the average atomic size within the matrix. This reduction in atomic size causes the Mg lattice to expand, a phenomenon known as lattice dilation [22]. This lattice expansion can influence the alloy’s mechanical properties by affecting dislocation movement and solid solution strengthening mechanisms.

**Figure 2:**
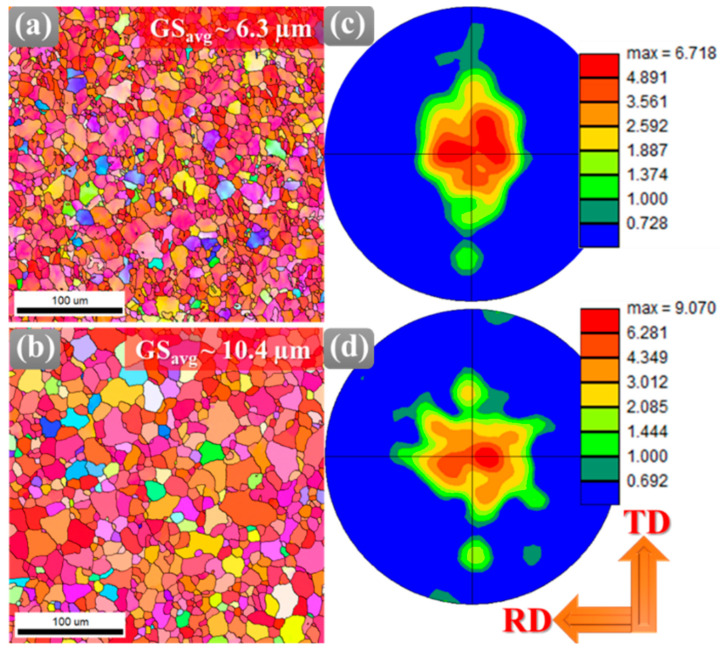
EBSD analysis showing (**a**,**b**) inverse pole figure (IPF) maps and corresponding (**c**,**d**) (0001) pole figures of the Mg-Al-Zn-Ca alloy in AR state and after HT, respectively.

Figure 2 shows the effect of post-annealing treatment on the microstructure and texture evolution. The inverse pole figure (IPF) maps presented in Figure 2a,b clearly demonstrate that the average grain size increased from 6.3 µm in the AR sample to 10.4 µm in the HT sample. This increase in grain size is attributed to thermally driven grain growth during annealing, which is primarily governed by the reduction in grain boundary energy. However, the observed coarsening is non-uniform, as evidenced by the simultaneous presence of relatively coarse grains alongside fine grains. Such heterogeneous grain growth arises from variations in grain boundary mobility that are strongly influenced by precipitate pinning effects.

The Al_2_Ca precipitate tends to segregate at grain boundaries, exerting a retarding force on the boundary migration. Consequently, some grain boundaries remain stabilized, retaining a fine-grained morphology, whereas boundaries having lesser precipitate interactions migrate more freely, allowing abnormal growth of certain grains [23]. In addition, the heterogeneity in stored dislocation energy from prior hot-rolling deformation promotes differential growth rates, where grains with higher stored energy preferentially expand during annealing. As a result, the overall microstructure of the HT sample reflects an increase in average grain size but with significant inhomogeneity, characterized by the coexistence of abnormally large grains embedded within a finer-grained matrix.

It is well known that when particles are sufficiently coarse and well spaced, particle-stimulated nucleation can dominate, leading to enhanced recrystallization [24]. The PSN mechanism greatly affects the texture evolution, which can be clearly observed from PFs shown in Figure 2c,d for AR and HT samples, respectively. For instance, the HT sample showed a dominant basal texture with a maximum intensity of ~9.1 m.r.d, higher than in the AR condition (~6.7 m.r.d.). Despite this maximum texture intensity, weaker texture components are observed surrounding the main basal orientation (Figure 2d). These secondary components likely originate from PSN near non-shearable Al_2_Ca precipitates, where newly recrystallized grains adopt more random orientations, and from minor rotations of grains during grain growth. Thus, the HT sample demonstrates that extensive recrystallization can coexist with texture strengthening when orientation-selective grain growth dominates.

### 3.2. Impact of Post-Annealing Treatment on Recrystallization Behavior

**Figure 3:**
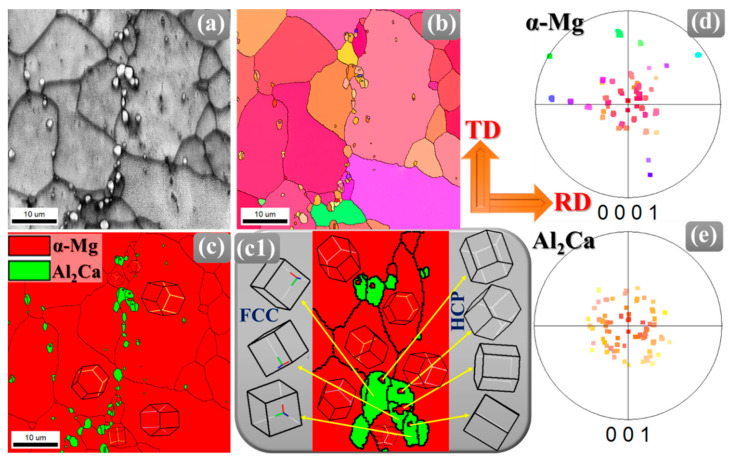
(**a**) IQ map, (**b**) IPF map, and (**c**) phase map of the HT sample. (**c1**) Magnified region from (**c**) showing various α-Mg and Al_2_Ca phase orientations. Discrete pole figures of (**d**) α-Mg and (**e**) Al_2_Ca precipitates are derived from (**b**).

Further magnified EBSD analysis has been performed on the HT specimen by acquiring a 50 µm × 50 µm map with a 0.02 step size to have a clear understanding of precipitate-grain boundary interaction on the recrystallization mechanism in a very localized area in detail (Figure 3). As Al_2_Ca has an FCC phase and α-Mg has an HCP phase, the partitioning has been made based on the phase for the detailed orientation analysis. It can be seen clearly from the image quality (IQ) maps that brighter particles are distributed along the grain boundaries instead of the grain matrix (Figure 3a). Moreover, the grains surrounded by a higher fraction of these precipitates are smaller compared to the ones having fewer precipitates at their grain boundaries (Figure 3b). Further phase map in Figure 3c confirmed the FCC phase nature of Al_2_Ca precipitates and the HCP phase nature of α-Mg. The precipitate-rich zones are further magnified in Figure 3(c1). Each grain and precipitate orientation is also marked.

A detailed examination of the precipitate-matrix interface in the HT sample, as shown in the magnified phase map of Figure 3(c1), provides key insight into the precipitation mechanism in the annealed sample. The schematic representation of the unit cells reveals a well-defined crystallographic orientation relationship between the FCC Al_2_Ca precipitates and the surrounding HCP α-Mg grains, indicating non-random interfacial alignment. This alignment minimizes interfacial free energy and enhances atomic coherency across the boundary, providing a thermodynamic driving force that governs the preferential orientation of the two phases. Such a structured relationship is characteristic of heterogeneous nucleation, in which the existing crystallographic planes of α-Mg act as favorable sites for Al_2_Ca precipitate settling. A closer analysis of the orientation behavior of the HCP α-Mg matrix and FCC Al_2_Ca precipitates, as illustrated in Figure 3d,e, reveals distinct crystallographic characteristics and alignment tendencies. In Figure 3d, the clustering of points near the basal poles indicates that most α-Mg grains maintain low-angle deviations from a primary texture component, consistent with limited intragranular misorientation. In contrast, the Al_2_Ca precipitates have a markedly different set of orientation clusters, as shown in Figure 3e. These clusters are not random but rather indicate that the FCC unit cells adopt specific orientation relationships relative to the surrounding HCP matrix. This confirms that Al_2_Ca precipitates preferentially align along certain crystallographic directions of the α-Mg matrix, such as aligning FCC {111} planes with HCP basal {0001} planes, which minimizes interfacial energy and promotes atomic coherency. Nevertheless, regions adjacent to Al_2_Ca precipitates display deviations from this primary basal texture.

These locally misoriented α-Mg grains arise from PSN, where strain fields around rigid particles generate new grains with orientations that are rotated relative to the bulk texture. The resulting high-angle boundaries (HAGBs) and weakly oriented grains contribute to a broader orientation distribution, manifested as dispersed points around the main basal pole or minor secondary clusters in the pole figure.

Consequently, while the majority of the matrix maintains favorable basal alignment, PSN by Al_2_Ca precipitates introduces localized heterogeneity, which can influence both the microstructural evolution and the orientation of precipitates in these regions. Importantly, this microstructural interplay also influences recrystallization. Second-phase particles, in combination with solute additions, modify grain boundary energetics through solute drag effects while controlling the kinetics of precipitate formation [25]. They promote nucleation via PSN and hinder grain growth through Zener pinning during recrystallization at the same time [26]. The distribution and size of Al_2_Ca particles generate localized deformation fields that facilitate new grain nucleation, while their pinning action limits excessive grain coarsening.

**Figure 4:**
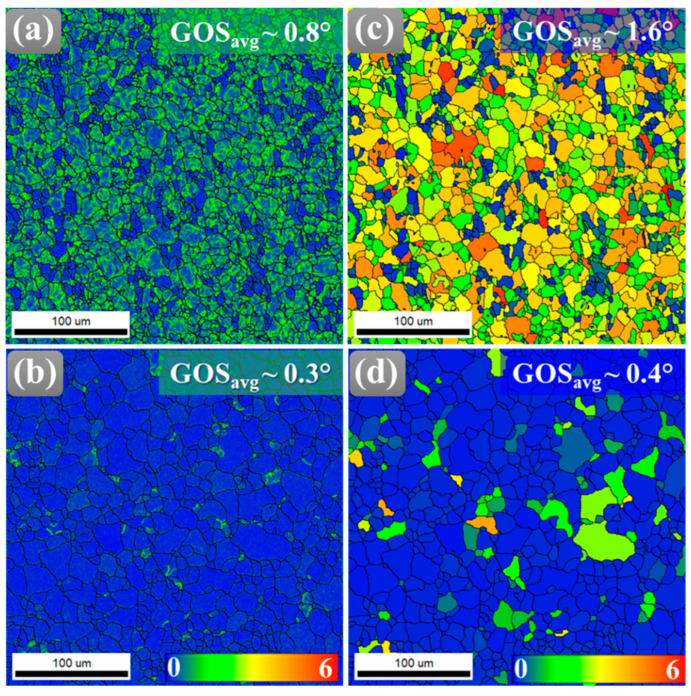
(**a**,**b**) kernel average misorientation (KAM) maps, (**c**,**d**) grain orientation spread (GOS) maps of the Mg-Al-Zn-Ca alloy in AR state and after HT, respectively.

Recrystallization is widely recognized as a fundamental mechanism for relieving localized stresses, leading to the formation of a microstructure with markedly reduced internal strain [27]. To quantitatively assess this stress relaxation and the associated microstructural heterogeneity, kernel average misorientation (KAM) analysis was applied (Figure 4). This method determines misorientation angles between neighboring points and estimates dislocation density by examining the first nearest neighbors within a maximum angular deviation of 5°, thereby providing a direct measure of local lattice distortion [28]. Here, Figure 4a,b show KAM maps with average KAM values for AR and HT samples, respectively. Figure 4a shows the greater strain accumulations within the grain matrix, which are homogeneously distributed throughout the microstructure of the AR sample. The higher KAM value (~0.8°) in the AR sample further reflects the high dislocation density induced by the initial hot rolling process. During primary thermomechanical processing, such as rolling, dislocation interactions and entanglements promote the formation of low-angle grain boundaries (LAGBs), which manifest as networks of dislocation walls and cellular substructures within the grains. After annealing, these entangled dislocations are largely annihilated or rearranged during recrystallization, releasing stored strain energy, thus forming strain-free grains with the lowest KAM value (~0.3°).

Similarly, grain orientation spread (GOS) serves as a critical metric for quantifying intragranular misorientation uniformity. It directly reflects strain heterogeneity within grains and provides insight into the recovery mechanisms that reduce lattice distortion during annealing. In the AR sample, elevated GOS values arise from plastic strain accommodation, which generates complex dislocation substructures and pronounced lattice curvature. This results in a high average misorientation relative to the mean grain orientation. By contrast, the HT sample exhibits a pronounced reduction in GOS, as newly formed recrystallized grains possess nearly uniform internal orientations (Figure 4d). The transition from a high, non-uniform GOS distribution to a low, homogeneous one quantitatively demonstrates the replacement of deformed grains with strain-free counterparts, signifying the progression of recrystallization and the release of stored strain energy.

The role of Al_2_Ca precipitates is particularly significant in this process. As thermally stable, non-shearable particles, they induce localized strain fields that hinder dislocation glide, promoting the accumulation and rearrangement of dislocations into LAGBs. During annealing, these LAGBs progressively absorb additional dislocations and rotate, transforming into HAGBs that define recrystallized grains [29,30]. The high stored energy concentrated in deformation zones surrounding the particles further accelerates boundary migration, thereby enhancing subgrain growth and driving the completion of recrystallization [31].

**Figure 5:**
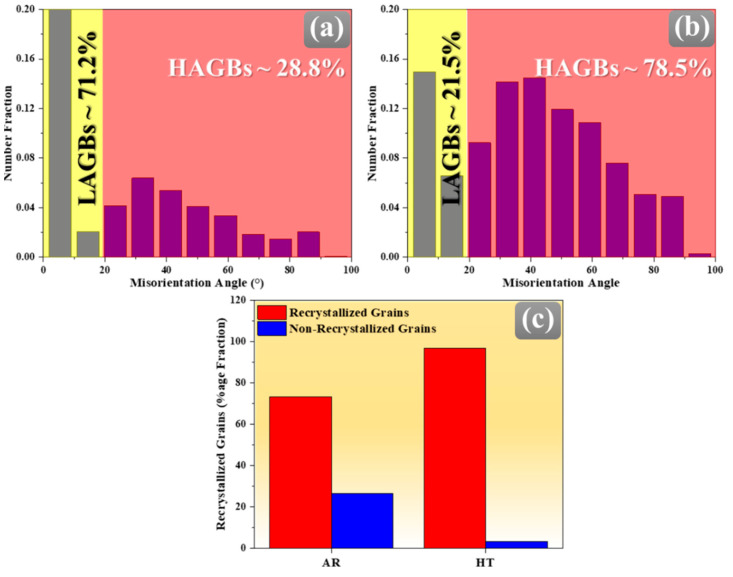
(**a**,**b**) Misorientation angle distribution profiles and (**c**) bar chart showing variation in the fraction of recrystallized and non-recrystallized grains in AR and HT specimens of Mg-Al-Zn-Ca alloys, respectively.

This interpretation is further corroborated by the misorientation angle distribution profiles of AR and HT samples, illustrated in Figure 5. The fraction of HAGBs increased significantly in the HT sample compared to the AR sample, demonstrating the transformation of LAGBs into HAGBs through subgrain rotation and PSN-assisted nucleation during static recrystallization. The higher proportion of HAGBs in the HT specimen reflects the replacement of subgrain boundaries with fully developed high-angle boundaries, indicating extensive strain accommodation and lattice rearrangement. This is further reinforced by the bar chart in Figure 5c, which presents the fraction of recrystallized grains in each sample, determined based on the grain orientation spread (GOS < 2°) criterion [32]. It is evident from the analysis that the HT sample contains a substantially higher proportion of recrystallized grains, while the fraction of non-recrystallized grains is significantly reduced compared to the AR condition. Such evolution demonstrates that elevated thermal exposure during annealing promotes recovery, subgrain coalescence, and PSN, thereby facilitating the formation of new strain-free grains. Although the LAGB-to-HAGB transition is more commonly reported in DRX, similar evolution can also occur during static recrystallization via subgrain rotation in conjunction with the PSN effect.

### 3.3. Impact of Post-Annealing Treatment on the Mechanical Behavior

**Figure 6:**
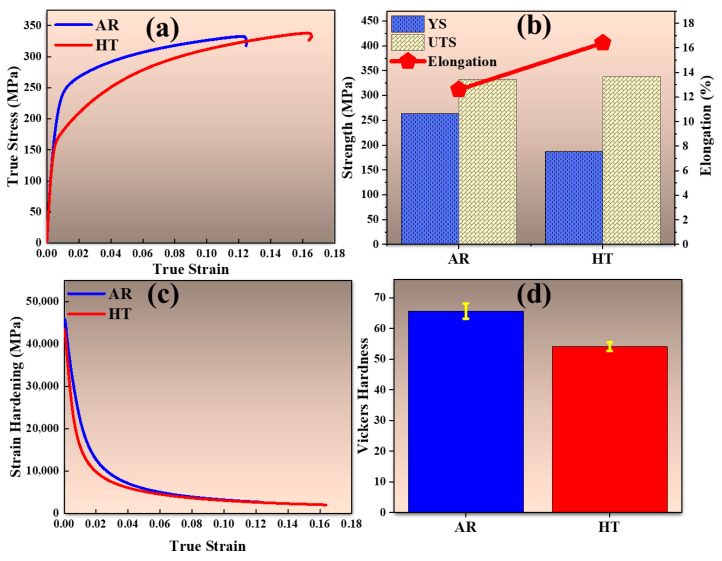
(**a**) True stress–strain curves, (**b**) bar chart showing comparison of strengths and elongation, (**c**) strain hardening behavior, and (**d**) Vickers hardness of AR and HT samples of Mg-Al-Zn-Ca alloys.

The true stress–strain curves of the AR and HT samples of Mg-Al-Zn-Ca alloy are presented in Figure 6a. The HT sample exhibits higher elongation and ultimate tensile strength (UTS), whereas the AR sample retains a higher yield strength (YS). Specifically, the YS decreased from 263 MPa in the AR state to 187.4 MPa after annealing, while the UTS slightly increased from ~332 MPa to ~338 MPa, as highlighted in the bar graphs in Figure 6b.

The elongation also improved significantly, increasing from 12.6% in the AR sample to 16.4% in the HT condition. The strain hardening rate curves in Figure 6c further show that the AR sample maintains a higher strain hardening rate throughout deformation, indicative of greater resistance to dislocation motion and enhanced strengthening during plastic flow. In contrast, the HT sample displays a reduced strain hardening rate, reflecting the microstructural softening and increased ease of dislocation movement following heat treatment. Similarly, the hardness results in Figure 6d reveal a decrease from 65.7 HV for the AR sample to 54.1 HV after annealing, indicating overall softening associated with the heat treatment. This reduction in Vickers hardness is closely linked to the lower dislocation density and relaxation of residual stresses induced by recrystallization, which also accounts for the drop in YS.

The observed property changes are closely linked to the underlying microstructural evolution. Post-annealing treatment triggered recrystallization [33], which was strongly facilitated by the PSN effect [15,34]. Here, second-phase particles acted as nucleation sites, leading to the development of equiaxed strain-free grains compared to the strain-hardened grains characteristic of the AR condition [26,35]. The kinetics of recrystallization were governed by the nucleation of Al_2_Ca precipitates, which in turn influenced the mechanical behavior [36]. This homogeneous microstructure reduced localized stress concentrations and provided additional pathways for dislocation motion, thereby improving the alloy’s ability to sustain more plastic deformation [37]. At the same time, the significant drop in YS and hardness can be attributed to the annihilation of dislocations and the relaxation of residual stresses during recrystallization, which lowered the resistance to plastic deformation [38]. Therefore, the mechanical response of the HT sample Mg-Al-Zn-Ca alloy reflects the combined effects of recrystallization-induced dislocation annihilation and microstructural homogenization. While yield strength and hardness decreased due to stress relaxation, the formation of fine, equiaxed grains and weakened texture enhanced ductility and slightly increased UTS. These changes highlight the critical role of recrystallization in balancing strength and plasticity, enabling simultaneous improvement in formability and overall mechanical performance.

## 4. Conclusions

This study demonstrates that post-annealing treatment profoundly modifies the microstructure, texture, and mechanical performance of the Mg-3Al-1Zn-1Ca alloy through a complex interplay between recrystallization and precipitate-boundary interactions. The Al_2_Ca exerted a dual influence by promoting recrystallization via PSN and restricting grain growth through Zener pinning. EBSD-based orientation analyses revealed that annealing facilitated extensive recrystallization, reduced dislocation density, and converted low-angle boundaries into high-angle boundaries, thereby relieving internal stresses and refining the overall microstructure. Concurrently, texture analysis indicated the emergence of stronger basal components, accompanied by weaker secondary orientations introduced by PSN. Mechanically, annealing led to a reduction in yield strength and hardness, reflecting the annihilation of dislocations and stress relaxation, while ultimate tensile strength remained nearly unaffected, and ductility was significantly improved. This effectively demonstrates the role of post-annealing treatment in improving the formability of the hot-rolled alloy while retaining adequate load-bearing capacity.

## Data Availability

The original contributions presented in this study are included in the article. Further inquiries can be directed to the corresponding author.

## Abbreviations

The following abbreviations are used in this manuscript:

AR	As received
HT	Heat-treated
IPF	Inverse pole figure
PSN	Particle simulated nucleation
EBSD	Electron backscattered diffraction
GOS	Grain orientation spread
KAM	Kernel average misorientation
